# Editorial

**Published:** 1841-07-24

**Authors:** 


### MEDICAL EXAMINER.

DEVOTED TO MEDICINE, SURGERY, AND THE COLLATERAL SCIENCES.

No. 30.] PHILADELPHIA, SATURDAY, JULY 24, 1841. [Vol. IV.

### THE MEDICAL EXAMINER.

PHILADELPHIA, JULY 24, 1841.

### MEDICAL REFORM.

To the Editors of the Medical Examiner.

Gentlemen,

I notice with pleasure the discussion upon which you have entered of the important subject of Medical Reform. The mere agitation of the question must, I think, be attended with good results; and, I am quite sure that the views which you have thrown out are shared by the mass of the profession. But, gentlemen, while you are proposing to raise the standard of qualification for the privilege to practice medicine, is it not notorious that, easily as that privilege is now obtained, it may be, and is, dispensed with in the successful attempt to reach the profits, and even the honours of the profession? In your own city, are not some of the most successful and eminent practitioners, (eminent in the estimation even of the educated and intelligent,) to be found in the ranks of those who laugh degrees to scorn, and will continue to pursue their career of lucky charlatanism, careless of the difficulties which you may choose to interpose in the acquirement of a diploma, the want of which they do not feel. Will you not then, gentlemen, while proceeding with the topic of Medical Reform, say a few words upon another which bears closely upon the former—*the suppression of quackery*—and oblige, among others, your constant reader,

Medico-Chirurgus.

*Richmond*, (Va.) July 21st, 1841.

The subject of quackery is of course, as our correspondent suggests, involved in the question of Medical Reform. It is naturally asked, why attempt to improve our system of medical education, so long as we are refused protection from the competition of the entirely uneducated? Our correspondent says, why raise the standard of qualification for licences to practice, when we see them altogether dispensed with? Indeed, the spectacle of successful quackery around us has shaken the faith of many in the whole system of universities and diplomas, and some even would throw the business of the physician entirely open, leaving it to be regulated by the ordinary laws of supply and demand. These are points upon which we intended entering during the present discussion, and we take the occasion of the communication before us to express our views now. We think that the public safety requires from the practitioner of medicine a guarantee that he is master of his profession. In other avocations, the public can appreciate the skill and talents of those they employ, while they can, to a certain extent only, judge of these qualities in a physician. We think then, that the public has a right to be presented with a certified list of educated persons, to whom, in emergencies, they may safely entrust their lives. So far, and so far only, would we carry the system of examinations and licences. We would divest it of every feature of monopoly. After taking care that the public should *know the best*, we would leave them free to *pursue the worst*. By improving the system of regular education, physicians will offer the best opposition to the progress of quackery. The real value of degrees in medicine will be accurately appreciated by the public; nor can it be doubted that the ease with which they are now obtained serves to depreciate them in popular estimation. From this very increasing quackery, we deduce an argument in favour of Medical Reform. Let us demand a higher scale of qualification from those who ask for the privilege of a degree, and the degree itself will have a higher value in the public eye. The more strongly marked is the line that separates the regular practitioner from the quack, the lower must the latter sink.

